# Impact of Social Media Use on Mental Health, Academic Performance, and Procrastination Among Medical College Students in Aruba

**DOI:** 10.7759/cureus.108583

**Published:** 2026-05-10

**Authors:** David Khanimov, Arnaw Kishore, Allen J Izraliov

**Affiliations:** 1 Department of Emergency Medicine, Xavier University School of Medicine, Oranjestad, ABW; 2 Department of Microbiology, East Point College of Medical Sciences and Research Centre, Bengaluru, IND

**Keywords:** anxiety, educational status, medical student, procrastination, social media

## Abstract

Background

Social media platforms (SMPs) have become an integral part of students' lives, influencing learning behaviors, time management, and mental well-being. However, there is limited evidence regarding their impact among medical students in Aruba. This study aimed to evaluate social media usage patterns and examine their perceived effects on mental health, academic performance, and academic procrastination.

Methods

A cross-sectional online survey was conducted between April and July 2025 among 25 medical students at Xavier University School of Medicine using a structured 29-item questionnaire. Participants were recruited through convenience sampling via voluntary online participation. Descriptive statistics were reported as frequencies and percentages. Due to the small sample size, Fisher's exact test was used to assess associations between social media usage patterns (platform and duration) and outcomes including mental health, academic performance, and procrastination. Pearson correlation analysis was performed to evaluate relationships between daily usage (hours/day) and academic score, perceived academic performance, and procrastination. A p-value of <0.05 was considered statistically significant. The small sample size is an important limitation and may limit the generalizability of the findings.

Results

Participants classified as heavy social media users (>3 hours/day; n=16/25 (64%)) reported higher levels of anxiety (66.7% vs. 22.2%; p=0.041) and greater mental or physical health impacts (55.6% vs. 11.1%; p=0.040) compared to light users (≤3 hours/day). They also demonstrated significantly higher academic procrastination, including increased distraction while studying (66.7% vs. 22.2%; p=0.041) and a greater likelihood of missing academic deadlines (55.6% vs. 11.1%; p=0.040). Although heavy users reported a stronger urge to use social media (61.1% vs. 22.2%), this difference was not statistically significant (p=0.097). Light users were significantly more likely to use social media for academic purposes (77.8% vs. 22.2%; p=0.017). Correlation analysis showed that increased time spent on social media was significantly negatively associated with academic score (r=-0.47; p=0.015) and perceived academic performance (r=-0.44; p=0.019). A weak positive correlation was observed between social media use and academic procrastination (r=0.17), which was not statistically significant (p=0.41).

Conclusion

Moderate social media use may support academic engagement, particularly when used for educational purposes; however, excessive use is associated with increased anxiety, poorer well-being, greater academic procrastination, and lower academic performance. These findings suggest that the impact of social media depends not only on duration but also on the purpose and pattern of use. Promoting mindful, structured, and balanced social media habits may help optimize students' academic outcomes and well-being.

## Introduction

The upsurge of social media platforms (SMPs) has dramatically changed how students communicate, share information, and interact with peers socially across the globe [1]. There were approximately 5.66 billion social media users worldwide in October 2025, representing 73.2% of the global population [2]. Among young adults, medical students demonstrate particularly high engagement levels, often accessing platforms multiple times daily [3-5]. Studies have reported that SMPs play a dual role, facilitating academic exchange among peers while also providing entertainment and social connectivity for stress relief [4,5].

Prior research examining the relationship between social media use and academic outcomes has shown mixed results. Some studies report educational benefits, including access to instructional videos, research updates, and collaborative study discussions, whereas others associate excessive or passive use with psychological distress, reduced attention, increased procrastination, and lower academic performance [3,6,7]. Social media dependence, characterized by compulsive and excessive use that interferes with daily responsibilities, has emerged as a growing concern within medical education [8].

Several studies from the Caribbean report high levels of social media and internet use among university students; however, the relationship between social media dependence and academic performance remains complex and heterogeneous [9,10]. Some investigations document negative associations between problematic social media use and academic achievement, including lower grade point averages and self-reported academic decline [11-13]. Conversely, other studies report neutral or even positive perceptions of academic benefit [3,14]. These inconsistencies may reflect differences in usage patterns, measurement instruments, cultural contexts, and sampling methodologies [10-12].

Recent literature suggests that cognitive engagement may mediate the relationship between social media usage and academic performance. When social media use promotes active cognitive processing and knowledge construction, academic benefits may emerge; however, passive consumption and distraction-driven use may impair learning outcomes [15].

In Aruba, digital penetration remains high, with approximately 88,000 social media users (81.4% of the total population) reported in October 2025, of whom approximately 81% use Facebook [16,17]. However, limited evidence exists regarding social media use among medical students in Aruba. To our knowledge, this study represents the first cross-sectional evaluation of social media usage patterns and their association with mental health, academic performance, and academic procrastination among medical students at Xavier University School of Medicine. The primary aim of this study is to evaluate usage patterns and assess their perceived impact on psychological well-being, academic outcomes, and procrastination. Additionally, the study analyzes the correlation between daily usage duration with students' academic score, perceived academic performance, and procrastination. Understanding this relationship may provide insight into promoting balanced and mindful social media use within medical education.

## Materials and methods

Study design

This quantitative cross-sectional study was conducted between April 2025 and July 2025 at Xavier University School of Medicine, located in Oranjestad, Aruba. The study aimed to evaluate SMP use patterns and their effect on mental health, academic performance, and academic procrastination.

Ethical consideration

Participation was entirely voluntary, and all participants received a clear explanation of the purpose of the study prior to involvement. Informed consent was obtained from each respondent, and strict measures were implemented to ensure confidentiality and anonymity throughout the study. Ethical approval was obtained from the Research Ethics Committee of Xavier University School of Medicine before conducting the study (approval number: XUSOM/RC/MD/24-25/012).

Research instrument

A self-administered, English-structured questionnaire (see Appendices) was developed to assess social media usage patterns and explore their association with mental health, academic performance, and academic procrastination among medical students. The questionnaire consisted of 29 items divided into five sections: (1) socio-demographic information, (2) patterns of social media use, (3) social media dependence and mental well-being, (4) academic performance, and (5) academic procrastination. Data from sections 1 and 2 were collected based on multiple-choice questions. Other sections were rated on a five-point Likert scale from 1=strongly disagree, 2=disagree, 3=neutral, 4=agree, and 5=strongly agree. For analysis, responses were later collapsed into three categories (disagreement, neutral, and agreement) to facilitate statistical comparison, given the small sample size. This study was designed as an exploratory institutional survey using a concise structured questionnaire to minimize respondent burden and improve participation within a small student population. Selected items addressing academic procrastination were included based on commonly reported behaviors in published literature, rather than applying a full standardized scale. However, this may limit comparability with studies using a standardized psychometric tool.

Inclusion and exclusion criteria

Students who were currently enrolled in pre-medical and medical courses in college and actively using social media were eligible to participate. All participants of the age group 18-45 years were included in the study. Students who did not have access to the internet to complete online surveys or who were on leave, were suspended, or had withdrawn from the program were excluded from the study.

Data collection and analysis

Questionnaires were distributed electronically using Google Forms (Google LLC, Mountain View, California, United States) to ensure accessibility. Data entry and management were performed using Microsoft Excel, Version 2019 (Microsoft Corporation, Redmond, Washington, United States), and statistical analyses were conducted using IBM SPSS Statistics for Windows, Version 26.0 (IBM Corp., Armonk, New York, United States). Based on prior research indicating that usage of social media more than three hours per day is associated with increased risks of mental fatigue, academic distraction, and reduced productivity, the present study classified >3 hours/day of social media user as heavy users and those who spent ≤3 hours/day as light users.

Content validity was assessed by a panel of experts, who evaluated the items for relevance, clarity, and comprehensiveness. Minor modifications were made based on their feedback. Face validity was established by pre-testing the questionnaire among a small group of medical students (n=10) to ensure clarity and ease of understanding. The study sample (n=25) also served as a pilot to assess feasibility. Internal consistency of the questionnaire was evaluated using Cronbach's alpha.

Descriptive statistics were used to summarize categorical variables as frequencies and percentages. Fisher's exact test was applied to assess the association between social media usage duration (≤3 hours/day vs. >3 hours/day) and categorical outcomes, because of the small sample size. Pearson correlation analysis was used to evaluate the relationship between daily social media use (hours/day) and academic outcomes. The correlation coefficient (r) and p-value were reportedly applicable. A p-value of <0.05 was considered statistically significant.

Sample size consideration

As this was an exploratory cross-sectional study conducted within a single medical college, all eligible students who consented during the study period were invited to participate. A total of 27 students responded to the survey, of whom 25 met the inclusion criteria and were included in the final analysis. Due to the limited size of the eligible student population at the institution, a convenience sampling approach was adopted rather than a priori statistical sample size calculation.

## Results

Socio-demographic characteristics

A total of 27 students responded to the questionnaire, of whom 25, who met the inclusion criteria, were included in the study. The demographic distribution of the 25 study participants shows that students aged 18-25 constituted the largest group (n=11; 44%), followed by other categories, each with seven participants (28% each). The study included nine male and 16 female participants (1:1.8 ratio). Female students were slightly more represented across all age groups, particularly in the 25-35 range. The majority of participants were medical students (n=23; 92%), while only two were enrolled in the pre-medical program (Table 1). Reliability analysis was performed and indicated high internal consistency across all groups (Cronbach's alpha=0.87). 

**Table 1:** Distribution of the study participant students based on demographic characteristics Percentage was calculated based on n=25; findings should be interpreted cautiously because of the limited sample size.

	Number of participants (%)	Female participants	Male participants
Age (years)			
18-25	11 (44%)	7	4
25-35	7 (28%)	5	2
35-45	7 (28%)	4	3
Course of study			
Medical	23 (92%)	15	8
Pre-medical	2 (8%)	1	1

Pattern of SMP use practices

**Figure 1:**
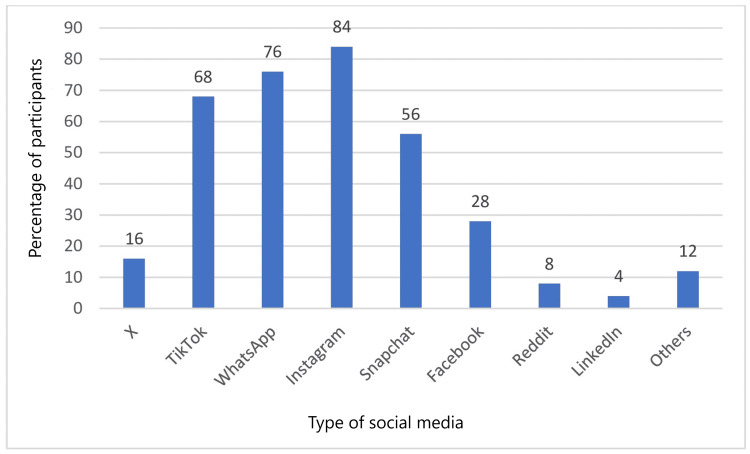
Percent distribution of the study participants based on their SMP preferences SMP: social media platform

The majority of students reported active use of multiple SMPs, with Instagram (84%), WhatsApp (76%), and TikTok (68%) being the most frequently used (Figure 1). The main reported purposes of social media use were entertainment (n=20; 80%), social networking (n=17; 68%), and educational activities (n=16; 64%), and these data are presented in Table 2. Most participants (60%; n=15) used social media for 3-4 hours daily, while 20% (n=5) used it for 1-2 hours, 16% (n=4) for 2-3 hours, and only 4% (n=1) for more than four hours per day. During class, 52% (n=13) reported using social media rarely, 24% (n=6) sometimes, 20% (n=5) never, and 4% (n=1) often. Additionally, more than half of the respondents (56%; n=14) used more than three different SMPs, while 44% (n=11) used three or fewer, indicating a broad and active digital engagement among students. 

**Table 2:** Distribution of the study participants based on SMP use practices Heavy users were defined as participants reporting >3 hours/day of social media use (combined 3-4 hours/day and >4 hours/day categories), while light users were defined as participants reporting social media use ≤3 hours/day. SMP: social media platform

	Number of participants (%)
Time spent on social media	
1-2 hours	5 (20%)
2-3 hours	4 (16%)
3-4 hours	15 (60%)
More than 4 hours	1 (4%)
Usage of social media during class	
Rarely	13 (52%)
Sometimes	6 (24%)
Never	5 (20%)
Often	1 (4%)
Number of social media used	
Students using ≤3 types of social media	11 (44%)
Students using >3 types of social media	14 (56%)
Purpose of social media	
Entertainment	20 (80%)
Social network	17 (68%)
Educational activities	16 (64%)

Psychological impacts of social media

The study found that heavy social media users demonstrated stronger dependence on social media compared to light users. While a significantly higher proportion of heavy users reported feeling anxious or troubled when unable to access social media (66.7% vs. 22.2%; p=0.041), a strong urge to use social media was also observed among heavy users (61.1% vs. 22.2%; p=0.097), though this difference was not statistically significant. Heavy users further reported significantly higher mental and physical health impacts (55.6% vs. 11.1%; p=0.040), as well as relationship strain (55.6% vs. 22.2%; p=0.208). Although not statistically significant, a greater tendency to use SMPs as an escape from personal or academic stress (55.6% vs. 33.3%; p=0.411) and more failed attempts to reduce usage (50% vs. 33.3%; p=0.677) were noted (Table 3). 

**Table 3:** Comparison of perceived positive response of heavy users and light users Light users were defined as students reporting social media use ≤3 hours/day (n=9), while heavy users were defined as students reporting >3 hours/day (combined 3-4 hours/day and >4 hours/day categories; n=16). Fisher's exact test was used to compare categorical responses between the two groups because of the small sample size. A p-value of <0.05 was considered statistically significant.

No.	Item	Comparison summary	Light user (n=9) agreed response (%)	Heavy user (n=16) agreed response (%)	P-value	Total agreed %
1	Strong urge to use social media	Heavy users showed a significantly stronger urge to use social media	22.2%	61.1%	0.097	44%
2	Use social media as an escape	More heavy users reported using social media to cope with stress or challenges, though not significant	33.3%	55.6%	0.218	44%
3	Failed attempts to reduce use	Heavy users tended to show lower self-control with more failed attempts to reduce use	33.3%	50%	0.311	44%
4	Anxiety when unable to use	Heavy users experienced significantly more anxiety when unable to use social media	22.2%	66.7%	0.041	48%
5	Think/planning social media use	Heavy users were more preoccupied with social media use	33.3%	61.1%	0.148	48%
6	Use for academics	Light users used social media more for academic purposes than heavy users	77.8%	22.2%	0.017	44%
7	Social media improves performance	Heavy users more often perceived an academic benefit, though not significant	66.7%	55.6%	0.691	60%
8	Share academic knowledge	Heavy users slightly more likely to share knowledge via social media	77.8%	66.7%	1.000	72%
9	Supplement learning	Both groups reported similar use of social media for learning	77.8%	61.1%	0.661	68%
10	Dependence of academic success	Perceived dependence on social media for performance was moderate and similar across groups	66.7%	50%	0.677	56%
11	Attempted to discontinue	Heavy users more often attempted and failed to discontinue social media	22.2%	55.6%	0.208	40%
12	Delay projects	Procrastination was higher among heavy users	33.3%	55.6%	0.411	44%
13	Aware but fails to act	Awareness of responsibilities was similar, but heavy users acted less	33.3%	33.3%	1.000	36%
14	Distracted while studying	Heavy users were significantly more distracted during study sessions	22.2%	66.7%	0.041	48%
15	Postpone assignments	Both groups showed procrastination, slightly higher among heavy users	33.3%	61.1%	0.228	48%
16	Miss deadlines	Heavy users missed deadlines more frequently, significantly so	11.1%	55.6%	0.041	36%
17	Aware of negative effects	Awareness of negative effects was higher among light users	77.8%	27.8%	0.017	52%
18	Personal relationships affected	Heavy users more often reported relationship strain	22.2%	55.6%	0.208	40%
19	Health impacted	Heavy users significantly reported more mental/physical health impacts	11.1%	55.6%	0.040	36%
20	Dependence on social media	Heavy users felt more dependent on social media	22.2%	61.1%	0.097	44%
21	Stress when not checking	Heavy users had significantly higher stress when unable to check notifications	22.2%	66.7%	0.041	48%

Impact of social media on academic performance

Light users were more likely to use social media for academic purposes (77.8% vs. 22.2%; p=0.017), suggesting more purposeful and academically oriented engagement. Although not statistically significant, heavy users were somewhat more inclined to perceive that social media improves their academic performance (55.6% vs. 66.7%; p=0.691) and to share academic knowledge with classmates (66.7% vs. 77.8%; p=1.000). Similarly, both groups reported comparable tendencies to supplement classroom learning using educational content on social media (61.1% vs. 77.8%; p=0.661), and their perceived dependence of academic success on social media was moderate and not significantly different (50% vs. 66.7%; p=0.677) (Table 3). Pearson correlation results indicated statistically significant negative correlation between the per-day use of SMPs and academic score (r=-0.47; p=0.015), concluding that students who spent more time on social media tended to report lower academic performance scores.

Impact of social media on academic procrastination

Dependence of academic success on social media was moderate and similar across both groups (66.7% vs. 50%; p=0.677). While awareness of social media's negative effects was significantly greater among light users (77.8% vs. 27.8%; p=0.017), heavy users more frequently attempted but failed to discontinue social media (55.6% vs. 22.2%; p=0.208) and showed higher tendencies to delay academic projects (55.6% vs. 33.3%; p=0.411). Awareness of responsibilities was comparable, though heavy users acted on them less often (33.3% vs. 33.3%; p=1.000). A significant difference was found in distraction while studying (66.7% vs. 22.2%; p=0.041), where heavy users were markedly more prone to interruptions by social media. Similarly, missing important academic deadlines was higher among heavy users (55.6% vs. 11.1%; p=0.040), reflecting poorer time management. Additionally, heavy users tended to postpone assignments more (61.1% vs. 33.3%; p=0.226) and felt greater dependence on social media overall (61.1% vs. 22.2%; p=0.097) (Table 3).

Correlation analysis

There was a significant moderate negative correlation between social media usage time and academic achievement score (r=-0.47; p<0.05), as well as between usage time and perceived academic performance (r=-0.44; p<0.05). However, the correlation between social media usage time and perceived academic procrastination was weak and not statistically significant (r=0.17; p>0.05) (Table 4). 

**Table 4:** Correlation between time of social media uses with academic benefits and procrastination A p-value of <0.05 was considered significant.

Relationship	Pearson (r)	P-value
Time vs. academic achievement	-0.47	0.015
Time vs. perceived academic performance	-0.44	0.019
Time vs. perceived academic procrastination	0.17	0.41

## Discussion

This study is among the first to investigate social media use and its effects on mental health, academic performance, and academic procrastination among medical students in Aruba. It demonstrates that the relationship between social media dependence and academic performance is multifaceted and influenced by both the duration and purpose of use. The findings indicate that Instagram is the most commonly used platform, consistent with research among university students in Greece reporting its popularity among younger age groups [18]. WhatsApp emerged as the second most used platform, although previous studies have identified it as the most dominant platform among undergraduates [19,20]. In contrast, Facebook was used by only a minority of students in this study, differing from earlier findings in the UAE and Kenya, where it was the leading platform [21,22]. These variations likely reflect temporal shifts in platform popularity, the emergence of new applications, and evolving user engagement patterns [19-22].

More than 60% of participants reported using social media for over three hours daily, which was classified as high-frequency use and aligns with thresholds used in studies from Sri Lanka and Jordan [20,23]. However, global definitions of problematic use vary, ranging from more than two to four hours per day [23-25]. Such discrepancies may be attributed to differences in cultural context, academic workload, and digital engagement habits, as well as rapid technological advancements that encourage prolonged use [23-27]. Most students reported accessing social media during leisure time rather than during classes, consistent with prior research [20,25]. Additionally, the widespread use of multiple platforms for entertainment, education, and social networking highlights the integral role of social media in students' daily academic and social lives [18,23-25].

The study identified key indicators of problematic social media use, including compulsive checking, difficulty reducing usage, preoccupation with online activity, anxiety when unable to access platforms, and the use of social media as a coping mechanism. These behaviors are consistent with maladaptive coping patterns described in previous literature [26]. Approximately 44% of participants exhibited such behaviors, with heavy users reporting significantly higher levels of compulsive use, anxiety, and unsuccessful attempts to reduce usage. These findings are consistent with studies conducted in India, Indonesia, and Saudi Arabia, which also reported negative associations between heavy social media use and mental well-being [19,27,28]. Importantly, these results suggest an association between prolonged use and compulsive engagement rather than a direct causal relationship [19,20].

The relationship between social media use and academic performance appears to be complex. While a substantial proportion of students perceived short-duration use as beneficial for academic purposes, increased time spent on social media was significantly associated with lower academic achievement and perceived academic performance. This finding aligns with prior research indicating that excessive digital media use may reduce study time, increase distraction, and impair sustained attention and is consistent with evidence from medical students globally [23-28]. Notably, around 70% of participants reported using social media for academic purposes, such as sharing educational content and supplementing classroom learning [25,26]. This dual role suggests that the academic impact of social media depends more on how it is used rather than solely on duration.

Several studies suggest that academically oriented use, such as participation in online educational groups and accessing instructional content, can enhance learning through improved information access and peer collaboration [10]. In contrast, entertainment-focused and excessive use has been associated with procrastination and poorer academic outcomes. In the present study, students using social media for less than three hours daily reported fewer procrastination behaviors, whereas those exceeding this duration experienced greater distraction and poorer time management. Similar findings have been reported in previous studies, where heavy users were more likely to engage in last-minute studying, resulting in increased stress and reduced learning quality. These patterns may negatively affect students' overall well-being and academic success [29,30].

Correlation analysis further supported a negative association between social media use and academic performance, consistent with the displacement hypothesis, whereby time spent on non-academic activities replaces time allocated to studying. However, the relationship between total time spent on social media and academic procrastination was weak and not statistically significant. This suggests that duration alone may not adequately capture maladaptive usage patterns. Instead, procrastination may be more strongly influenced by psychological factors such as self-regulation, motivation, and compulsive behaviors [21,23]. Overall, these findings indicate that while social media can serve as a valuable academic tool, excessive and unregulated use may interfere with concentration and time management, highlighting the need for targeted interventions such as digital wellness programs and strategies promoting mindful and purposeful technology use among students.

Limitations and future directions

The small sample size (n=25) and cross-sectional assessment at a single time point limited statistical power and prevented analysis of the long-term trend in medical students' social media use. As the data were collected using a self-administered questionnaire, recall bias and social desirability bias may have influenced the reporting of daily social media use, procrastination, and academic performance. In addition, an established rating tool was not employed for psychological assessment, which may limit comparability with studies using validated instruments. The single college convenience sample may also limit generalizability because of institutional and cultural specificity. Future research should use large multicentric samples, validation instruments, and longitudinal designs to better examine associations related to excessive social media use.

Clinical implications

These findings have important practical implications. Universities and mental health professionals should consider implementing targeted digital wellness interventions that promote mindful social media use, self-regulation, and effective time management strategies. The findings of this study suggest that excessive social media use may serve as a potential behavioral marker for adverse mental health outcomes and academic difficulties among medical students. Increased usage was associated with higher levels of anxiety and academic procrastination, highlighting the need for early identification and targeted interventions. Light users perceive social media as beneficial for academic purposes, suggesting the importance of promoting responsible and purposeful use of digital platforms. These findings support the integration of digital wellness education, time management strategies, and mental health support programs within medical curricula to mitigate the negative impact of excessive social media use.

## Conclusions

This study provides cross-sectional evidence on social media use and dependence-related behaviors among medical students in Aruba. Moderate social media use appears to support academic performance and achievements, particularly when used for educational purposes; however, excessive use is associated with increased anxiety, poorer well-being, greater academic procrastination, and reduced academic performance. Importantly, the findings suggest that the quality and purpose of social media use may be more influential than duration alone, as not all usage is detrimental. Promoting mindful, structured, and purpose-driven social media habits may help optimize academic outcomes while minimizing negative effects on students' well-being. However, these findings should be interpreted cautiously because a small sample size, single-institutional settings, and self-reported responses limited the study. Large multicentric studies using validated assessment tools are needed to confirm these observations.

## Appendices

Research survey questionnaire

Section 1: Socio-demographic Information

Q1. Gender Female Male

Q2. Age 18-25 years 25-35 years 35-45 years and above

Q3. Course of study Medical Pre-medical

Q4. Academic examination score (%) ≥85% 84%-70% 69%-55% ≤54%

Section 2: Patterns of Social Media Use

Q5. Time spent on social media per day 1-2 hours 2-3 hours 3-4 hours More than 4 hours

Q6. Usage of social media during class Never Rarely Sometimes Often

Q7. Types of social media platforms used (multiple responses allowed) Facebook X WhatsApp LinkedIn Snapchat Reddit Skype Instagram TikTok Others

Q8. Main purpose of social media use (multiple responses allowed) Entertainment Social networking Educational activities

Section 3: Social Media Dependence and Mental Well-Being

Q9. I feel a strong urge to use social media increasingly Strongly disagree Disagree Neutral Agree Strongly agree

Q10. I use social media as an escape from personal and academic challenges Strongly disagree Disagree Neutral Agree Strongly agree

Q11. I have made unsuccessful attempts to reduce my social media usage Strongly disagree Disagree Neutral Agree Strongly agree

Q12. I become anxious or troubled when unable to use social media Strongly disagree Disagree Neutral Agree Strongly agree

Q13. I spend considerable time thinking about or planning my social media use Strongly disagree Disagree Neutral Agree Strongly agree

Q14. Social media has affected my personal relationships Strongly disagree Disagree Neutral Agree Strongly agree

Q15. My physical or mental health has been impacted by social media use Strongly disagree Disagree Neutral Agree Strongly agree

Q16. I consider myself dependent on social media Strongly disagree Disagree Neutral Agree Strongly agree

Q17. I feel anxious or stressed when unable to check my social media notifications Strongly disagree Disagree Neutral Agree Strongly agree

Section 4: Academic Performance

Q18. I primarily use social media for academic purposes Strongly disagree Disagree Neutral Agree Strongly agree

Q19. Social media usage has positively influenced my academic performance Strongly disagree Disagree Neutral Agree Strongly agree

Q20. I utilize social media to share academic knowledge with classmates Strongly disagree Disagree Neutral Agree Strongly agree

Q21. I supplement classroom learning with educational content from social media Strongly disagree Disagree Neutral Agree Strongly agree

Q22. I believe my academic performance depends on social media use Strongly disagree Disagree Neutral Agree Strongly agree

Section 5: Academic Procrastination

Q23. I have attempted to temporarily discontinue social media use Strongly disagree Disagree Neutral Agree Strongly agree

Q24. I delay academic projects until the last minute Strongly disagree Disagree Neutral Agree Strongly agree

Q25. I am aware of my academic responsibilities but fail to act on them Strongly disagree Disagree Neutral Agree Strongly agree

Q26. I get easily distracted by social media when studying Strongly disagree Disagree Neutral Agree Strongly agree

Q27. I postpone assignments until they approach their deadline Strongly disagree Disagree Neutral Agree Strongly agree

Q28. I regularly miss important academic deadlines Strongly disagree Disagree Neutral Agree Strongly agree

Q29. I am knowledgeable about the negative effects of social media use Strongly disagree Disagree Neutral Agree Strongly agree

Questions Q9-Q29 used a five-point Likert scale (1=strongly disagree; 2=disagree; 3=neutral; 4=agree; 5=strongly agree). Questions Q7 and Q8 allowed multiple responses.
